# Advances in Genome Editing With CRISPR Systems and Transformation Technologies for Plant DNA Manipulation

**DOI:** 10.3389/fpls.2020.637159

**Published:** 2021-01-14

**Authors:** Satya Swathi Nadakuduti, Felix Enciso-Rodríguez

**Affiliations:** ^1^Department of Environmental Horticulture, University of Florida, Gainesville, FL, United States; ^2^Plant Molecular and Cellular Biology Program, University of Florida, Gainesville, FL, United States; ^3^Centro de Investigación Tibaitatá, Corporación Colombiana de Investigación Agropecuaria – Agrosavia, Mosquera, Colombia

**Keywords:** gene-editing, CRISPR-Cas9, Cas variants, base editors, prime editing, *Agrobacterium* transformation, tissue culture, nanotechnology

## Abstract

The year 2020 marks a decade since the first gene-edited plants were generated using homing endonucleases and zinc finger nucleases. The advent of CRISPR/Cas9 for gene-editing in 2012 was a major science breakthrough that revolutionized both basic and applied research in various organisms including plants and consequently honored with “The Nobel Prize in Chemistry, 2020.” CRISPR technology is a rapidly evolving field and multiple CRISPR-Cas derived reagents collectively offer a wide range of applications for gene-editing and beyond. While most of these technological advances are successfully adopted in plants to advance functional genomics research and development of innovative crops, others await optimization. One of the biggest bottlenecks in plant gene-editing has been the delivery of gene-editing reagents, since genetic transformation methods are only established in a limited number of species. Recently, alternative methods of delivering CRISPR reagents to plants are being explored. This review mainly focuses on the most recent advances in plant gene-editing including (1) the current Cas effectors and Cas variants with a wide target range, reduced size and increased specificity along with tissue specific genome editing tool kit (2) cytosine, adenine, and glycosylase base editors that can precisely install all possible transition and transversion mutations in target sites (3) prime editing that can directly copy the desired edit into target DNA by search and replace method and (4) CRISPR delivery mechanisms for plant gene-editing that bypass tissue culture and regeneration procedures including *de novo* meristem induction, delivery using viral vectors and prospects of nanotechnology-based approaches.

## Introduction

The year 2020 marks a decade since the first gene-edited plants were generated using homing endonucleases and zinc finger nucleases by traditional *Agrobacterium* mediated genetic transformation (Gao et al., 2010; Osakabe et al., 2010; Zhang et al., 2010). Subsequently, TALENs have been developed and successfully shown to engineer plants (Cermak et al., 2011; Li et al., 2012). While initial gene-editing platforms are mostly protein-based DNA targeting systems, the discovery of guide RNA directed CRISPR/Cas9 revolutionized gene-editing because of its simplicity of use and versatility, replacing previous platforms, as one article termed genome-editing B.C. (Before CRISPR) (Urnov, 2018). There has been tremendous progress in genome engineering of various biological systems including plants using CRISPR systems, and it continues to be a rapidly evolving field. CRISPR technology not only caters to genome manipulation needs but is also re-purposed for a multitude of applications beyond genome editing (Adli, 2018). Less than a decade since the discovery of CRISPR/Cas9 as a method for genome editing, the year 2020 also marked awarding “The Nobel Prize in Chemistry” jointly to Dr. Emmanuelle Charpentier and Dr. Jennifer A. Doudna (The Nobel prize press release)^1^. The first CRISPR edited plants were developed in 2013 (Li et al., 2013; Shan et al., 2013) and since then this technology has been applied in 45 plant genera across 24 families (Shan et al., 2020). Recognizing gene-editing as a modern breeding tool, the regulatory landscape of genetically modified crops in the United States and several parts of the world has been revised (Nadakuduti et al., 2018).

This review will focus on the various classes of CRISPR-Cas derived gene-editing reagents that have been recently added to the CRISPR tool kit including: (1) Cas effectors and multiple Cas variants expanding the range of target sites and increased specificity along with tissue specific genome editing tool kit, (2) base editing for precisely installing all 12 possible base pair conversions without any DNA double stranded breaks (DSBs) or donor templates (3) prime editing that can copy the information on guide RNA directly into the target DNA site, all of which combinedly offer multitude of applications in genome editing and beyond. Plant cells have unique challenges for delivering the gene-editing reagents compared to other organisms, including the presence of a rigid cell wall, frequency of recalcitrant species not amenable to genetic transformation, common occurrence of polyploidy and integration of Cas9 expression cassettes into the host genomes to name a few. In addition to CRISPR-Cas reagents, this article will also focus on recent innovations in delivering these reagents to plants, the existing gaps, and future perspectives.

## CRISPR-Cas Nucleases and Variants Expand the Range of Target Site Recognition and Lower the Reagent Delivery Load for Plant Genome Editing

CRISPR-Cas9 nuclease belongs to class 2, type-II CRISPR systems which are RNA-guided endonucleases that generate blunt DSB at the genomic DNA target site. A CRISPR RNA (crRNA) and a *trans-*activating crRNA (tracrRNA) are fused into a single guide RNA (sgRNA) molecule that directs the Cas9 nuclease (Jinek et al., 2012). The most used Cas9 derived from *Streptococcus pyogenes* (SpCas9) requires a protospacer adjacent motif (PAM) sequence of “NGG” in the target DNA sequence. Cas9 has two nuclease domains: the HNH domain cleaves the guide-RNA bound complementary target DNA strand whereas the RuvC-like domain cleaves PAM-containing non-complementary DNA strand thereby generating a DSB, 3 bp upstream of PAM within the protospacer sequence (Barrangou et al., 2007; Jinek et al., 2012).

One of the limitations of CRISPR/Cas9 system is the “NGG” PAM requirement, reducing target recognition sites. A comprehensive list of Cas9 variants used in genome editing applications has been reviewed earlier (Anzalone et al., 2020). Some of the Cas9 variants including SpCas9-VQR, SpCas9-EQR, Cas9-NG, and xCas9 3.7 with PAM requirements of NGA, NGAG, NG, and NG/GAA/GAT, respectively, have been successfully used in plant species including, *Physcomitrella*, Arabidopsis, rice, tomato, and potato (Zhang et al., 2019). Furthermore, The Cas9 orthologs from *Staphylococcus aureus* (SaCas9) and *Streptococcus thermophilus* (St1Cas9) which recognize PAM sites NNGRRT and NNGGGT, respectively, have also been used successfully in Arabidopsis, potato, tobacco, rice, and citrus with relatively high editing efficiencies (Steinert et al., 2015, 2017; Kaya et al., 2016; Jia et al., 2017; Veillet et al., 2020).

Cas12 nucleases belong to class 2, type-V CRISPR systems which are mostly guided by a single crRNA (∼42 nt) compared to the Cas9 guide RNA (∼100 nt). Cas12 effectors lack HNH domain but possess only RuvC-like domain that can cleave both strands of the DNA target site generating a staggered cut with a 4–5 nt 5′ overhang (Zetsche et al., 2015). The most used Cas12 variant used for gene-editing in plants is LbCas12a that recognizes a T-rich PAM “TTTV” (Zhang et al., 2019). Furthermore, engineered variants of Cas12a with increased activities and target ranges have also been developed (Kleinstiver et al., 2019).

Another recent addition to the CRISPR toolbox is CRISPR-CasΦ, a hypercompact type-V CRISPR system comprising of a single CasΦ protein of ∼70-kilodalton that is about half the size of Cas9 or Cas12a. CRISPR-CasΦ is also a crRNA-guided dsDNA targeting nuclease with a minimal PAM requirement of 5′-TBN-3′ (where B = G, T, or C). Similar to Cas12a, CasΦ also does not require a tracrRNA and generates a staggered cut with 5′-overhangs (Pausch et al., 2020). CasΦ has been shown to be active in plant cells when delivered as ribonucleoproteins (RNPs) into Arabidopsis protoplasts editing *Phytoene desaturase* (*PDS*) gene, albeit with a low editing efficiency of 0.85% (Pausch et al., 2020). Furthermore, CRISPR – tissue specific knockout system (TSKO) established in Arabidopsis enables specific somatic gene knockouts in varied plants cell types/tissues by driving the Cas9 expression using cell type specific promoters (Decaestecker et al., 2019; Ali et al., 2020). The limitations in using tissue specific promoters, however, could be leaky expression and limited number of such promoters characterized thus far. TSKO can be further beneficial when expanded to other plant species and by identification of additional tissue specific promoters.

## Cytosine, Adenine, and Glycosylase Base Editors Capable of All Combinations of Precise Base Conversions Without Requiring DNA Double Stranded Breaks

**FIGURE 1:**
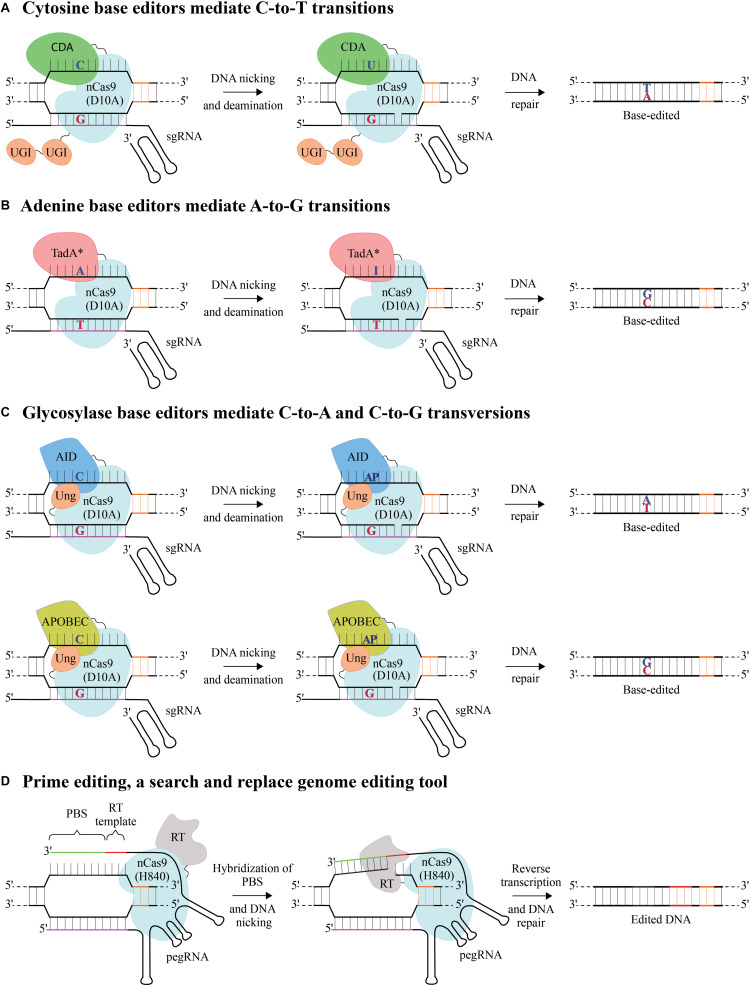
Base editing and prime editing using CRISPR systems **(A)** Cytosine base editors (CBE) mediate C-to-T conversion by using a nickase, nCas9 (D10A) fused to a cytidine deaminase (CDA) and uracil glycosylase inhibitor (UGI). After target DNA binding by sgRNA:nCas9 complex and formation of a single stranded R-loop, CDA catalyzes the conversion of cytosine (C) within the R-loop window in the PAM containing non-target strand to uracil (U) which has base-pairing properties of thymine (T). The UGI domain blocks the uracil DNA glycosylase (Ung) to catalyze U removal and initiate base excision repair thereby preventing U:G mismatch from being repaired back to a C:G. nCas9 generates a nick in the target DNA strand preferentially mediating a U:G mismatch to a T:A conversion. **(B)** Adenine base editors (ABE) mediate A-to-G conversion by using a nCas9 (D10A) fused to an evolved DNA processing adenosine deaminase (TadA*) which catalyzes the deamination of adenosine (A) to inosine (I) within the R-loop. I base pairs with C and read as G after DNA repair or replication. **(C)** Glycosylase base editors (GBE) mediate transversion mutations, C-to-A or C-to-G by using a nCas9 (D10A) fused to an activation-induced cytidine deaminase (AID) or APOBEC and Ung. After target DNA binding, nCas9 generates a nick in the target DNA strand and C is deaminated to U mediated by AID or APOBEC in non-target strand, Ung initiates the DNA repair by excising U and creating an abasic site (AP), enabling respective nucleotide conversions. **(D)** Prime editing uses an engineered reverse transcriptase (RT) fused to a nickase, nCas9 (H840A) that nicks the non-target strand of DNA and a prime editing guide RNA (pegRNA), which contains a 3′ RT template (Red) containing the required edits and primer binding sequence (PBS, green). The PAM containing non-target DNA strand is nicked, which then hybridizes to the PBS of the pegRNA and RT generates complementary DNA by copying the RT template in 3′ pegRNA to incorporate the desired mutations into the nicked DNA strand. 5′ spacer sequence in the guide RNA is in purple and a protospacer adjacent motif (PAM) in orange.

Base editors precisely convert one target DNA nucleotide to another using a catalytically impaired dead Cas9, dCas9 (D10A and H840A) or mostly using a nickase, nCas9 (D10A). Individual nicks generated by base editors are repaired by a more precise base excision repair pathway (BER) unlike the SpCas9 generated DSBs that are repaired typically by error prone non-homologous end joining (NHEJ) (Dianov and Hübscher, 2013; Ran et al., 2013), thereby minimizing the undesired byproducts due to gene-editing. Cytosine base editors (CBEs), catalyze C-to-T using a cytosine deaminase (CDA) – either rat APOBEC1/human activation induced cytidine deaminase (AID)/*Petromyzon marinus* CDA1, an AID ortholog (termed as target-AID) tethered to nCas9 (Figure 1A; Komor et al., 2016; Nishida et al., 2016). Adenine base editors (ABEs) catalyze A-to-G conversions using an evolved DNA processing deoxyadenosine deaminase (TadA^∗^) tethered to nCas9 (Figure 1B; Gaudelli et al., 2017). When the sgRNA binds to the target/complementary DNA strand to form an RNA-DNA hybrid, the PAM containing DNA strand is displaced to form a DNA “R-loop” (Jiang et al., 2016). The base conversions are mediated by exploiting the single stranded nature of this R-loop, exposing, and making the DNA accessible to CDA or TadA^∗^. This process allows the conversion of the respective bases within the R-loop (transcriptional RNA/DNA hybrid), defined as base editing “activity window.” Both CBEs and ABEs have been optimized and utilized in various plant species (Shimatani et al., 2017; Zong et al., 2017; Shan and Voytas, 2018; Li et al., 2020). There have been tremendous efforts toward improving base editors with increased efficiency and purity of the edited product to minimize by-stander mutations (Anzalone et al., 2020), some of which remain to be utilized in plants.

Cytosine base editors and ABEs facilitate only transition mutations, from C-to-T and A-to-G, respectively. However, recently developed glycosylase base editors (GBEs) can mediate transversion mutations such as C-to-A and C-to-G, making it feasible for current base editors collectively to convert from any base to any other base in the DNA (Zhao et al., 2020). GBEs were developed on the hypothesis that uracil-DNA glycosylase (Ung) catalyzes the removal of uracil (U) from DNA that is formed by deamination of cytosine and initiates BER causing C-to-A conversions (Zhao et al., 2020). Ung-nCas9-AID specifically binds to the target DNA, AID cleaves the amine group from C generating a U, while Ung excises U creating abasic site (AP site), followed by DNA repair resulting in C-to-A editing events (Figure 1C). Ung prevents C-to-T conversions, which would occur because of UGI (Ung inhibitor), typically used in case of CBE editors (Figure 1A). Using APOBEC1-nCas9-Ung, C-to-G conversions were obtained within the activity window (Figure 1C), specifically at the 6th base within the protospacer sequence (counting base 1 from distal end of PAM) suitable for position specific editing (Zhao et al., 2020).

## Prime Editing, A Versatile Genome Editing Technology Based on Target Primed Reverse Transcription

Recently, a “search-and-replace” prime editing (PE) method has been developed to directly copy the desired edit incorporated within the guide RNA, without requiring DSBs or a donor DNA repair template (Anzalone et al., 2019). PE is a breakthrough technology that can generate targeted insertions or deletions, or directly install precise transition and transversion mutations at targeted genomic loci, making it a versatile tool. PE is based on target primed reverse transcription mechanism analogous to retrotransposons, carried out by (1) prime editor protein, a fusion between nickase nCas9 (H840A) and an engineered reverse transcriptase (RT) enzyme that generates complementary DNA from an RNA template (2) a prime editing guide RNA (pegRNA) that encodes the primer binding site (PBS) and RT template containing intended edits within a 3′ extension appended to the sgRNA scaffold that targets the DNA site (Figure 1D). When nCas9 nicks the PAM containing DNA strand, it hybridizes to the PBS of the pegRNA and the RT copies the genetic information present on the RT template into the target DNA site. PE2 incorporates five mutations in M-MLV RT (D200N/L603W/T330P/T306K/W313F) to improve editing efficiencies while, PE3 includes an additional sgRNA to nick the non-edited strand as well, 14–116 nucleotides away from pegRNA induced nick to minimize the DSBs. This additional nicking helps in directing the DNA repair machinery to favor the incorporation of the edit during the resolution of heteroduplex DNA (Anzalone et al., 2019). Tools for prime editing and pegRNA design have been developed that could be used irrespective of the species in study (Prime editing tools)^2^^,3^. Prime editing has been implemented in cereal crops (Butt et al., 2020; Lin et al., 2020; Tang et al., 2020; Xu et al., 2020) and is yet to be used in diverse plant species.

A possible advantage of prime editing is having fewer bystander mutations compared to base editing, especially when multiple Cs or As are present in the editing activity window. It is also less restricted by PAM availability than other methods such as HDR, NHEJ or base editing, since the PAM-to-edit distance on average could be >30 bp (Anzalone et al., 2019). However, there are a large suite of base editors developed with improved efficiency, product purity, and DNA specificity along with widespread applicability (Yu et al., 2020). While prime editing has the potential to replace base editors, this technology is still nascent and is typically less efficient compared with the current generation base-editing systems with superior on-target and off-target DNA editing profiles (Anzalone et al., 2020). Therefore, depending on various criteria for gene-editing including the desired edit, availability of PAMs, efficiency of editing and off-target/bystander mutations, one must choose a suitable editing strategy for specific applications.

## Developments in Delivery of Gene-Editing Reagents Into Plant Cells

Gene-editing relies on plant genetic transformation and regeneration procedures, which are major bottlenecks in many species. By far, the most used method of plant genetic transformation is *Agrobacterium*-mediated delivery, accomplished by incorporating the DNA to be delivered within its transfer T-DNA, which ultimately becomes incorporated within the plant genome. The other method commonly used in monocot species is by particle bombardment using a gene gun. Both methods cause random integration of DNA into plant genomes. Any foreign DNA incorporation within the host DNA is considered genetically modified and mandates regulatory oversight. Cell walls present in plant cells pose a unique challenge for delivery of gene-editing reagents compared to any other cells. Protoplasts, like animal cells with only plasma membranes, offer a non-transgenic genome editing possibility. Protoplast transfection with plasmids expressing the gene-editing reagents or RNPs and regeneration of entire plants from these cells has been possible in a few species (Woo et al., 2015; Andersson et al., 2018; González et al., 2020). However, entire plant regeneration from single-celled protoplasts involve tissue-culture procedures for prolonged periods of time resulting in frequent and undesirable somaclonal variation. A recent study analyzed the protoplast regenerants and identified aneuploidy and structural chromosomal changes that can compromise plant phenotype (Fossi et al., 2019). Therefore, new methods to overcome these problems, especially bypassing tissue culture methods, are invaluable.

## Gene-Editing by Expression of Developmental Regulators and *de novo* Meristem Induction in Plants

**FIGURE 2:**
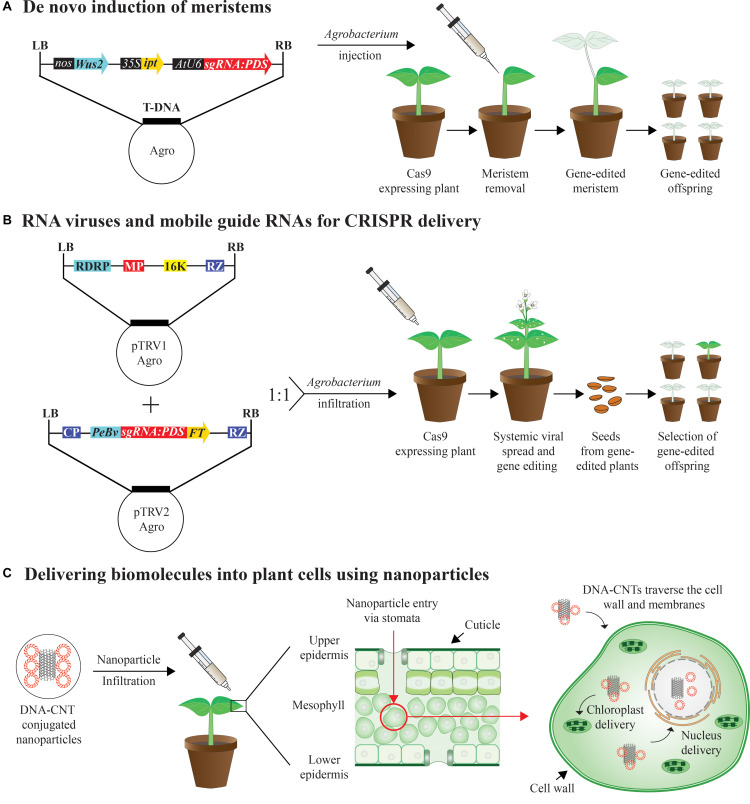
Breakthrough delivery methods for plant genome editing **(A)** *Agrobacterium tumefaciens* carrying expression cassette of the developmental regulators *Wuschel 2* (*Wus2*) driven by nopaline synthase (nos) promoter, *isopentenyl transferase* (*ipt*) driven by 35S promoter from the cauliflower mosaic virus (35S), and a single guide RNA driven by U6 promoter targeting the *Phytoene desaturase* (*PDS*) gene. A single guide RNA targeting *PDS* (sgRNA:PDS) is injected in Cas9 transgenic soil-grown plants with meristems removed. *pds* photobleaching phenotype is formed over time and transmitted to the next generation. **(B)** Tobacco rattle virus (TRV) is a bipartite, positive RNA virus with two genomes. While TRV1 harbors genes for replication, RNA-dependent RNA polymerase (RDRP), movement protein (MP), 16-kDa protein and terminating ribozyme (RZ), TRV2 has genes encoding for coat protein (CP) and manipulated to harbor sgRNA:PDS fused with *Flowering Locus T* (FT), a mobile RNA sequence at its 3′ end and driven by pea early browning virus subgenomic promoter (PeBV). TRV1 and TRV2 are delivered as T-DNA vectors via *Agrobacterium* and co-inoculated into leaves of Cas9 expressing plants. Systemic viral spread within the plant leads to photobleaching phenotype in the new growth in the plant. Germinated seedlings from the seeds of infiltrated plants also showed photobleaching indicating heritable gene-editing. **(C)** DNA- carbon nanotube (CNT) conjugates are delivered into surface of mature leaves using a needle-less syringe, enter through the stomates (red arrow), traverse the cell wall and cell membrane into the cytoplasm and delivery targeted to nucleus or to chloroplast can be achieved, where the cargo is released.

Developmental regulators (DRs) such as BABYBOOM (BBM) and WUCSHEL (WUS) upon transient expression have been previously shown to induce somatic embryogenesis in plants leading to genetic transformation of previously recalcitrant lines (Lowe et al., 2016). A similar approach has been used for gene-editing by inducing meristems in somatic cells by ectopically expressing DRs including BBM, WUS, SHOOT MERISTEMLESS (STM), and ISOPENTENYL TRANSFERASE (IPT) by *Agrobacterium* injection (Maher et al., 2020). Heritable gene-editing through this method has been achieved in *Nicotiana benthamiana* by transiently delivering guide RNAs and DRs to Cas9 overexpressing plants either by co-culturing seedlings germinated in liquid culture with *Agrobacterium* or by *Agrobacterium* injection of soil grown plants (Figure 2A; Maher et al., 2020). By having transgenic plants constitutively expressing Cas9, gene-editing using the *de novo* meristem induction method becomes a relatively high throughput method for gene-editing purposes mainly due to bypassing the time intensive tissue culture procedures. Furthermore, Growth-Regulating Factor 4 (GRF4) and its cofactor GRF-Interacting Factor 1 (GIF1) have been recently shown to increase the transformation frequencies in both monocots and dicots, most likely by regulating the cell proliferation and in the transition between stem cells to transit-amplifying cells. When GRF-GIF has been combined with CRISPR/Cas9 the frequency of genome-edited plants increased (Debernardi et al., 2020). Delivering Cas9 expression cassettes along with the sgRNA and growth regulators expressing cassettes via *Agrobacterium* into wild type plants is a feasible future approach which would facilitate DNA manipulation in a broad range of recalcitrant species.

## RNA Viruses and Mobile Guide RNAs for Heritable Plant Gene-Editing

Another heritable gene-editing method that has the potential of being a high-throughput method is by using a positive strand RNA virus, like the tobacco rattle virus (TRV) to deliver the sgRNAs into Cas9 over-expressing plants via *Agrobacterium* infiltration (Ellison et al., 2020). To achieve systemic gene-editing with heritable mutations, the sgRNAs have been fused with RNA mobile elements such as *Flowering locus T* (*FT*) to promote mobility of reagents to apical meristems, inducing germ line mutations. These modified sgRNAs are cloned into TRV vector which is delivered into plants by *Agrobacterium* infiltration (Figure 2B). This method has been shown to be efficient in generating heritable bi-allelic mutations with no evidence of virus transmission to progeny (Ellison et al., 2020). The drawback in utilizing positive strand RNA viruses or DNA viruses is their low cargo capacity, thereby preventing the delivery of entire CRISPR-Cas9 expression cassettes into plants. Recently, a negative strand DNA virus with larger cargo capacity called Sonchus yellow net rhabdovirus (SYNV) has been engineered to carry both SpCas9 and sgRNA sequences and delivered by *Agrobacterium* infiltration into wild type plants. All the mutations derived from M0 parents by this method were heritable (Ma et al., 2020).

## Nanoparticles for Delivering Biomolecules to Facilitate Plant Genome Engineering

Nanotechnology is an emerging field in agriculture and nano carriers present a unique opportunity for biomolecule delivery into plants and offer protection from degradation within the plant cells. Nano materials are defined as having at least one-dimension measure less than 100 nm. The plant cells possess hydrophilic cell walls which have a size exclusion limit of 5–20 nm, whereas that of the internal lipid plasma membrane is 500 nm (Cunningham et al., 2018; Landry and Mitter, 2019). Heavy metal nanoparticles (NP) are used for biolistic transformation where the cargo is delivered by means of force using a gene gun. However, single walled carbon nano tubes (CNTs) (∼1–1000 nm) and carbon dots (∼3 nm) can be chemically functionalized to carry genetic material and can diffuse through plant cell walls and deliver cargo to targeted cell organelles (Figure 2C). Recently, CNTs and carbon dots have enabled efficient DNA delivery into both nuclear (Demirer et al., 2019, 2020) and chloroplast genomes to achieve gene silencing (Kwak et al., 2019), without external biolistics or chemicals and with no DNA integration into mature plants. Nano carbons such as CNTs, fullerenes, graphene, and polymeric NPs including polyethyleneimine-coated NPs are promising for biomolecule delivery (DNA/RNA/Proteins and RNPs) into plant cells targeting germline or somatic tissues. The above-mentioned nano carriers have properties of cell-wall permeability and can be formulated and delivered into plant cells without using mechanical or chemical methods. Furthermore, these nano carriers protect the biomolecules from enzymatic degradation inside the cell, have low toxicity and facilitate attachment of specific ligands depending on the subcellular targets (Cunningham et al., 2018). Recent reviews focused on NP mediated plant genetic engineering further discuss the potential applications and limitations of this technology (Wang et al., 2019; Jat et al., 2020; Lv et al., 2020). In the near future, NP mediated delivery of gene-editing reagents into plant cells offers great potential to facilitate high throughput plant genome engineering.

## Potential Future Developments in the Field

The main goal in plant genome engineering is to get a beneficial phenotype through manipulating plant genomes to generate phenotype optimizing mutations. Ideally, to achieve this, we must have the ability to manipulate nucleotide sequences specifically and simultaneously at multiple sites in a genome irrespective of the plant species. Are we there yet? We now have reagents that can cater to a multitude of DNA manipulation possibilities and potential applications (Zhu et al., 2020) along with improved delivery mechanisms with no integration of transgenes. These advances also helped in re-designing the regulatory framework surrounding gene-edited crops. However, there are still challenges due to lack of editing efficiency, especially in polyploid crops, delivery limitations in certain plant species, occurrence of bystander and off-target mutations in the edited products (Jin et al., 2019). Direct delivery methods such as meristem induction and nanotechnology-based approaches offer opportunities for gene-editing in recalcitrant species. Reducing the cargo capacity further helps in the delivery process, which can be achieved by optimizing the CRISPR CasΦ system in plants. Further developments are anticipated in the fields of systems biology for high throughput and precise gene-editing, editing mitochondria or chloroplast genomes, editing plant genomes irrespective of species and without any integration of transgenes.

## Author Contributions

SN conceived the idea and wrote most of the manuscript. FE-R created figures and contributed to part of writing and overall improvement of the manuscript. Both authors read and approved the manuscript.

## Conflict of Interest

The authors declare that the research was conducted in the absence of any commercial or financial relationships that could be construed as a potential conflict of interest.

## Footnotes

1 https://www.nobelprize.org/prizes/chemistry/2020/press-release/

2 http://pegfinder.sidichenlab.org/

3 https://drugthatgene.pinellolab.partners.org/
